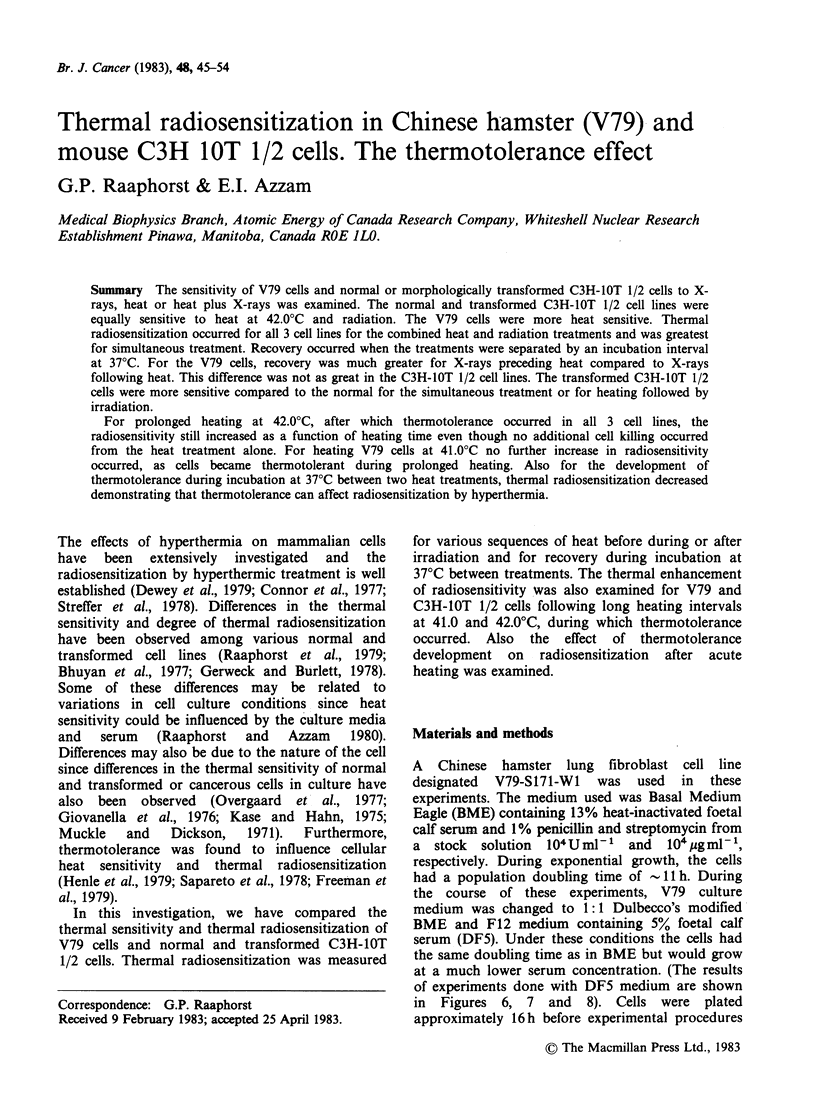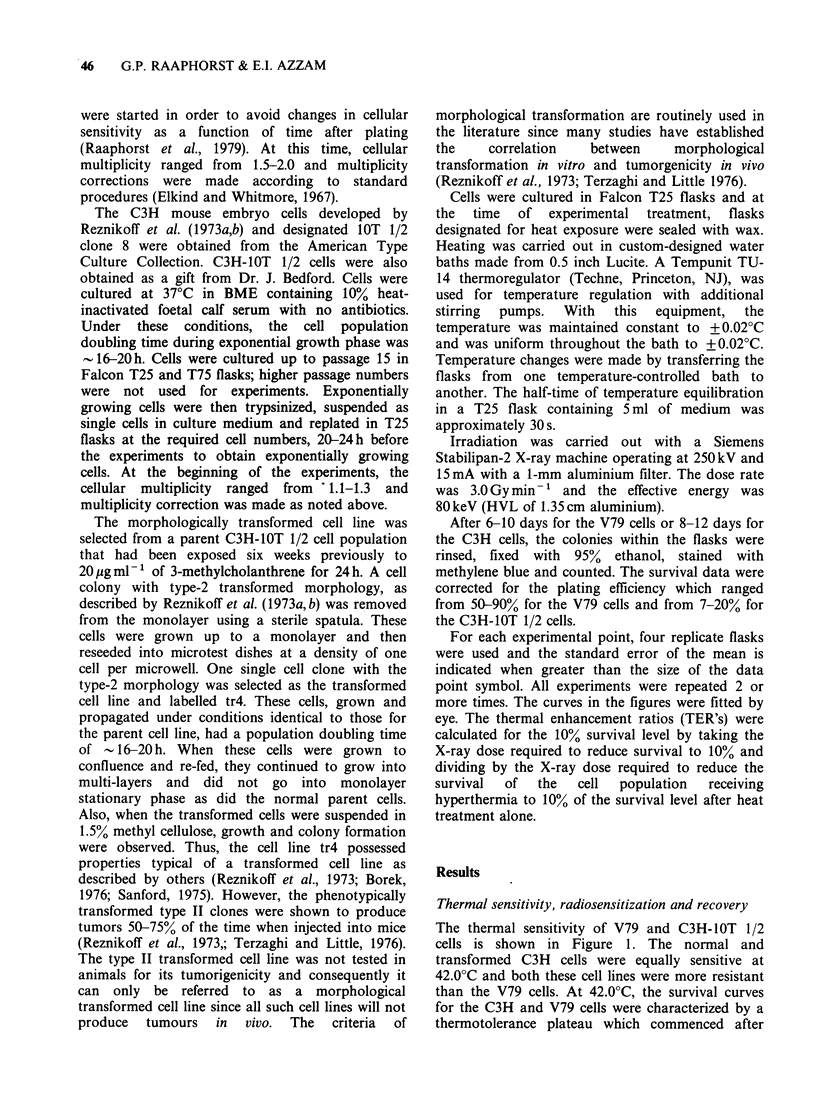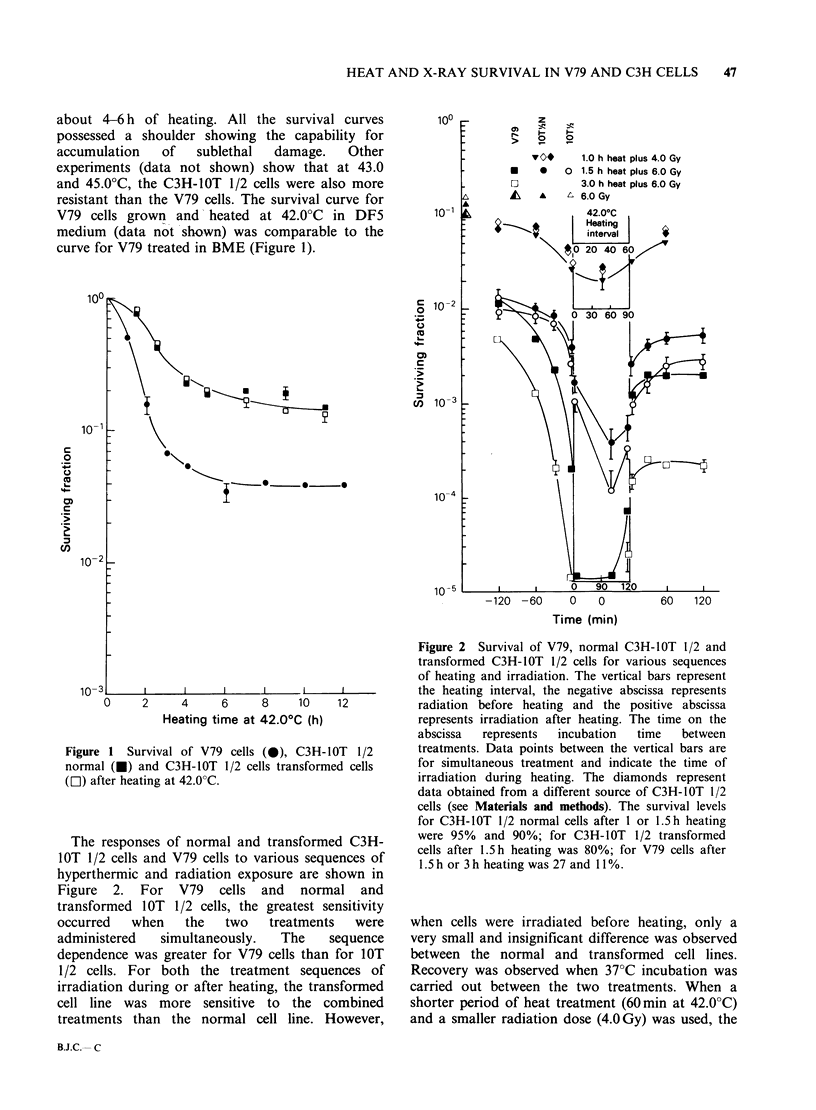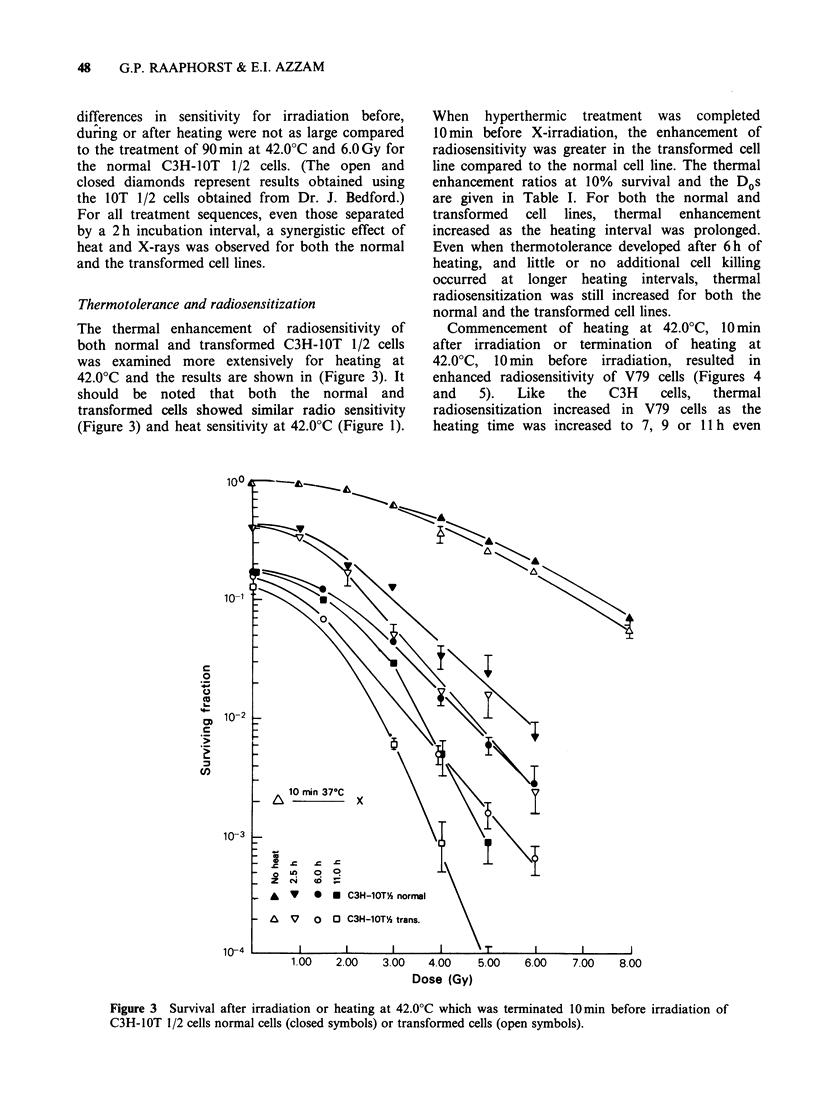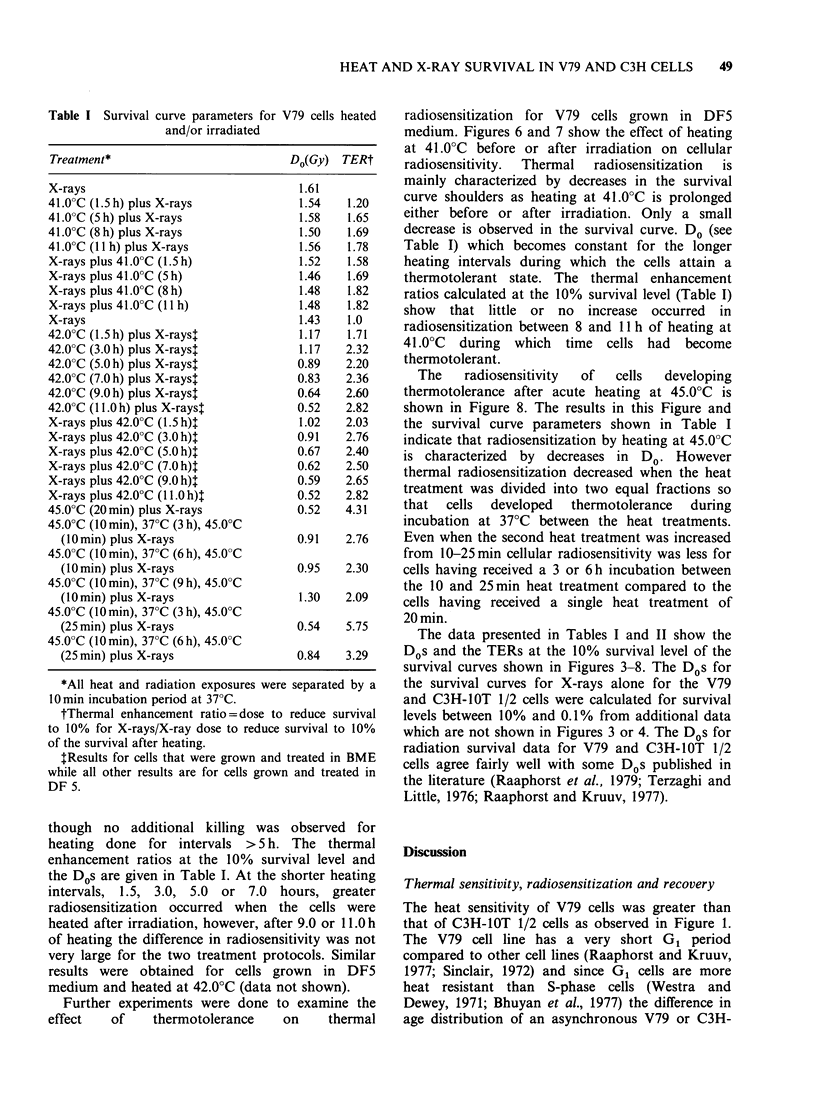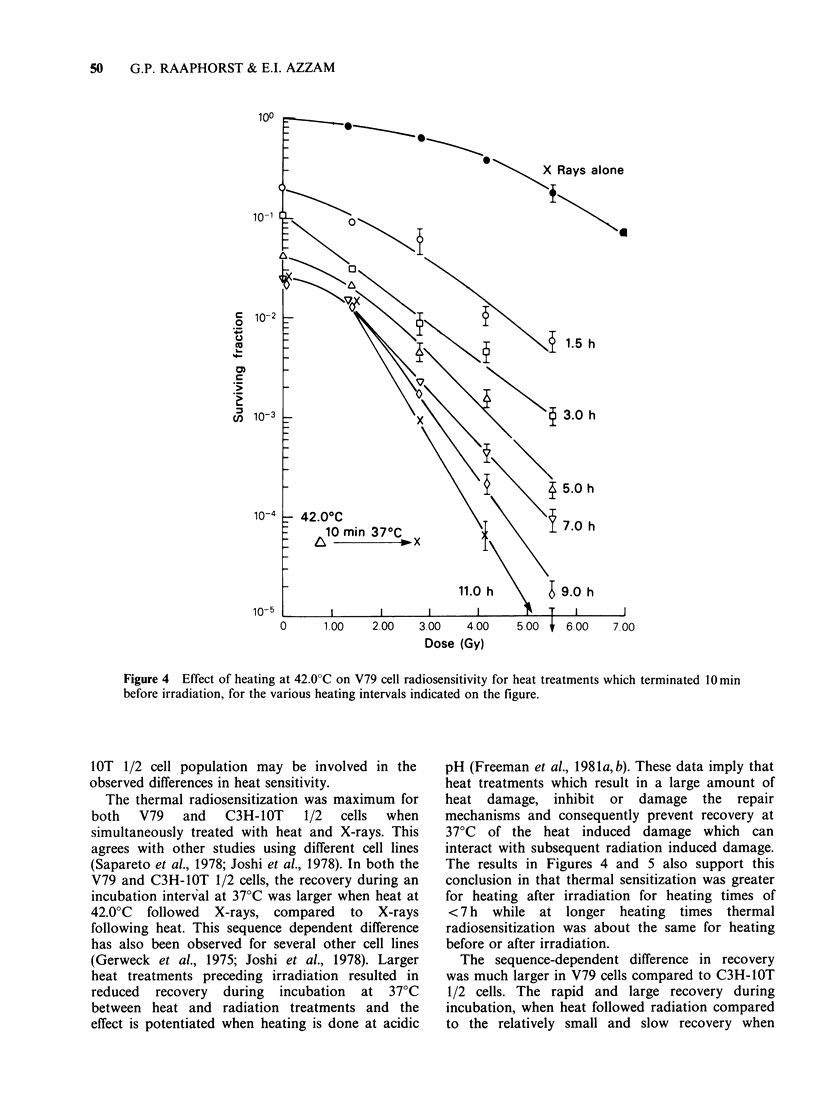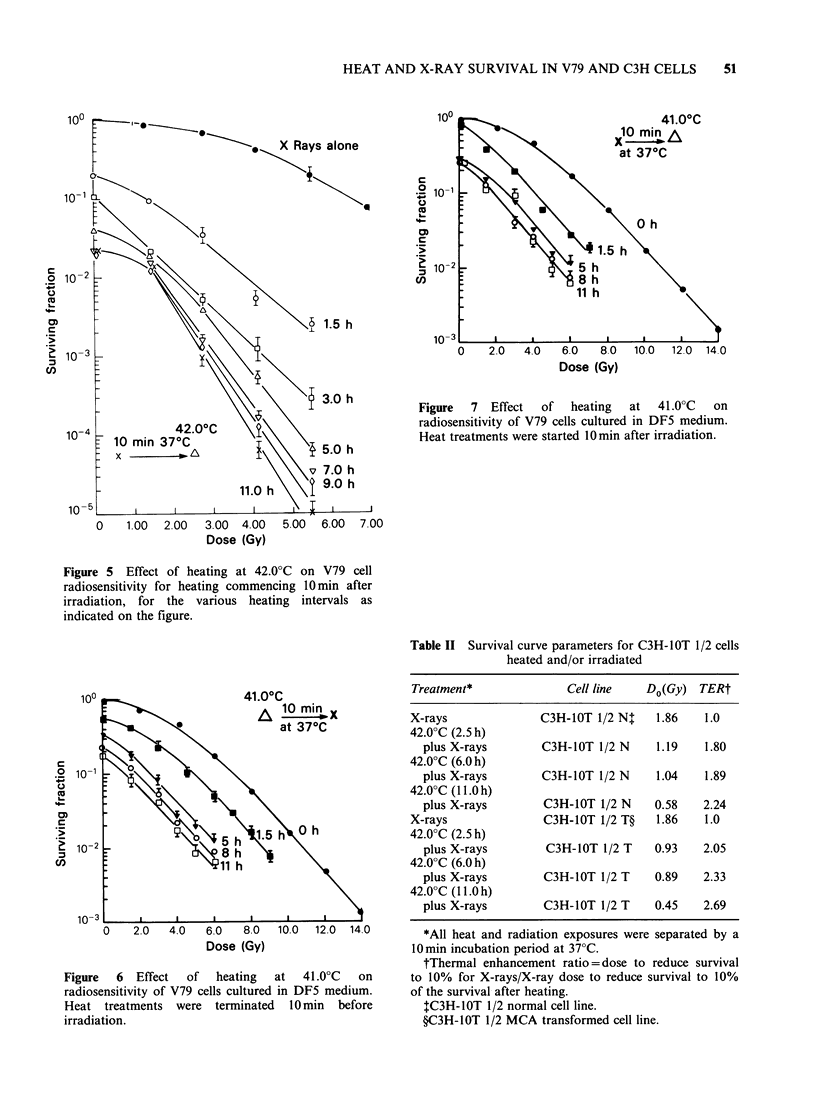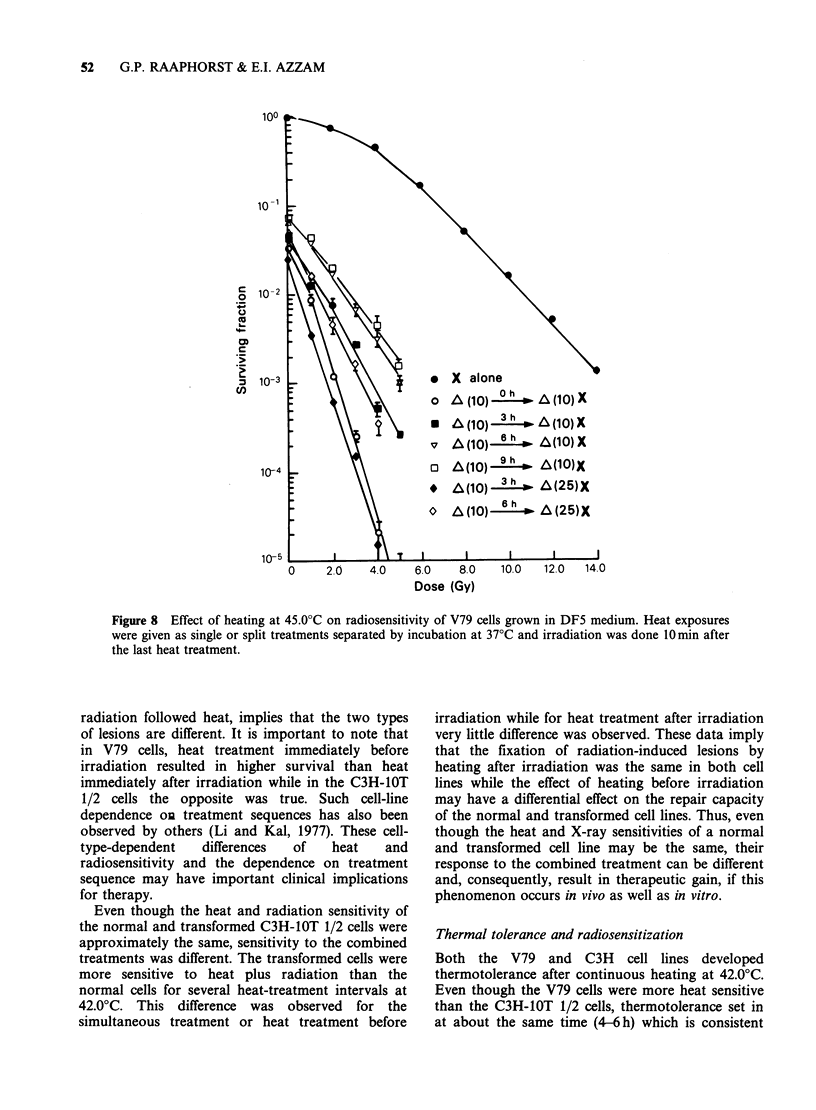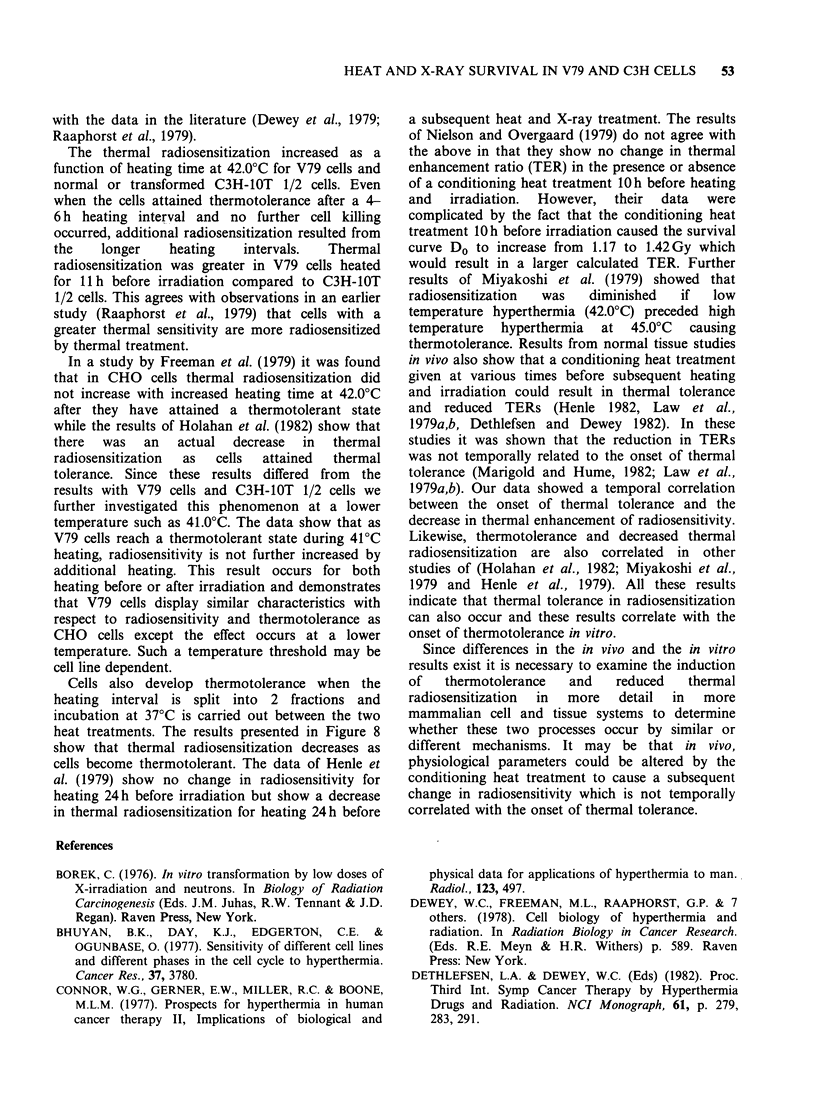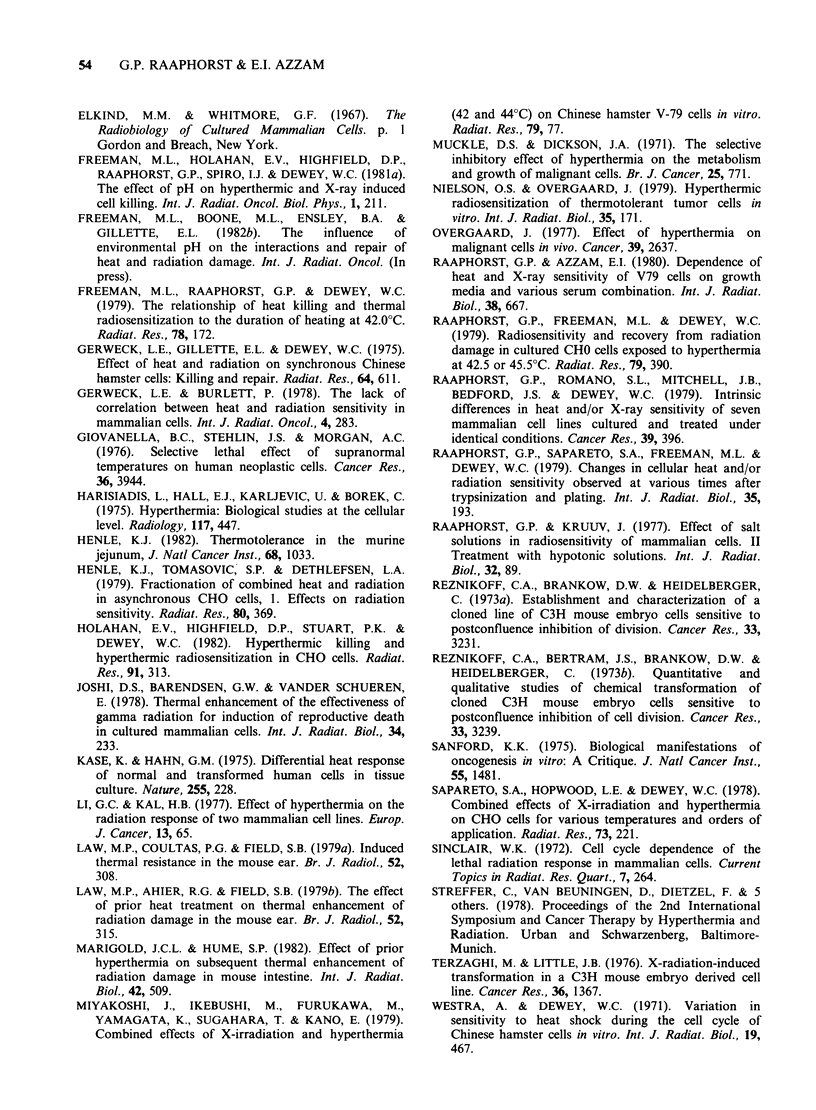# Thermal radiosensitization in Chinese hamster (V79) and mouse C3H 10T 1/2 cells. The thermotolerance effect.

**DOI:** 10.1038/bjc.1983.155

**Published:** 1983-07

**Authors:** G. P. Raaphorst, E. I. Azzam

## Abstract

The sensitivity of V79 cells and normal or morphologically transformed C3H-10T 1/2 cells to X-rays, heat or heat plus X-rays was examined. The normal and transformed C3H-10T 1/2 cell lines were equally sensitive to heat at 42.0 degrees C and radiation. The V79 cells were more heat sensitive. Thermal radiosensitization occurred for all 3 cell lines for the combined heat and radiation treatments and was greatest for simultaneous treatment. Recovery occurred when the treatments were separated by an incubation interval at 37 degrees C. For the V79 cells, recovery was much greater for X-rays preceding heat compared to X-rays following heat. This difference was not as great in the C3H-10T 1/2 cell lines. The transformed C3H-10T 1/2 cells were more sensitive compared to the normal for the simultaneous treatment or for heating followed by irradiation. For prolonged heating at 42.0 degrees C, after which thermotolerance occurred in all 3 cell lines, the radiosensitivity still increased as a function of heating time even though no additional cell killing occurred from the heat treatment alone. For heating V79 cells at 41.0 degrees C no further increase in radiosensitivity occurred, as cells became thermotolerant during prolonged heating. Also for the development of thermotolerance during incubation at 37 degrees C between two heat treatments, thermal radiosensitization decreased demonstrating that thermotolerance can affect radiosensitization by hyperthermia.


*Br. J. Cancer* (1983), **48**, 45–54

### Thermal radiosensitization in Chinese hamster (V79) and mouse C3H 10T 1/2 cells. The thermotolerance effect

G.P. Raaphorst & E.I. Azzam

*Medical Biophysics Branch, Atomic Energy of Canada Research Company, Whiteshell Nuclear Research Establishment Pinawa, Manitoba, Canada R0E 1L0.*

**Summary** The sensitivity of V79 cells and normal or morphologically transformed C3H-10T 1/2 cells to X-rays, heat or heat plus X-rays was examined. The normal and transformed C3H-10T 1/2 cell lines were equally sensitive to heat at 42.0°C and radiation. The V79 cells were more heat sensitive. Thermal radiosensitization occurred for all 3 cell lines for the combined heat and radiation treatments and was greatest for simultaneous treatment. Recovery occurred when the treatments were separated by an incubation interval at 37°C. For the V79 cells, recovery was much greater for X-rays preceding heat compared to X-rays following heat. This difference was not as great in the C3H-10T 1/2 cell lines. The transformed C3H-10T 1/2 cells were more sensitive compared to the normal for the simultaneous treatment or for heating followed by irradiation.

For prolonged heating at 42.0°C, after which thermotolerance occurred in all 3 cell lines, the radiosensitivity still increased as a function of heating time even though no additional cell killing occurred from the heat treatment alone. For heating V79 cells at 41.0°C no further increase in radiosensitivity occurred, as cells became thermotolerant during prolonged heating. Also for the development of thermotolerance during incubation at 37°C between two heat treatments, thermal radiosensitization decreased demonstrating that thermotolerance can affect radiosensitization by hyperthermia.

The effects of hyperthermia on mammalian cells have been extensively investigated and the radiosensitization by hyperthermic treatment is well established (Dewey *et al.*, 1979; Connor *et al.*, 1977; Streffer *et al.*, 1978). Differences in the thermal sensitivity and degree of thermal radiosensitization have been observed among various normal and transformed cell lines (Raaphorst *et al.*, 1979; Bhuyan *et al.*, 1977; Gerweck and Burlett, 1978). Some of these differences may be related to variations in cell culture conditions since heat sensitivity could be influenced by the culture media and serum (Raaphorst and Azzam 1980). Differences may also be due to the nature of the cell since differences in the thermal sensitivity of normal and transformed or cancerous cells in culture have also been observed (Overgaard *et al.*, 1977; Giovanella *et al.*, 1976; Kase and Hahn, 1975; Muckle and Dickson, 1971). Furthermore, thermotolerance was found to influence cellular heat sensitivity and thermal radiosensitization (Henle *et al.*, 1979; Sapareto *et al.*, 1978; Freeman *et al.*, 1979).

In this investigation, we have compared the thermal sensitivity and thermal radiosensitization of V79 cells and normal and transformed C3H-10T 1/2 cells. Thermal radiosensitization was measured for various sequences of heat before during or after irradiation and for recovery during incubation at 37°C between treatments. The thermal enhancement of radiosensitivity was also examined for V79 and C3H-10T 1/2 cells following long heating intervals at 41.0 and 42.0°C, during which thermotolerance occurred. Also the effect of thermotolerance development on radiosensitization after acute heating was examined.

Correspondence: G.P. Raaphorst
Received 9 February 1983; accepted 25 April 1983.

### Materials and methods

A Chinese hamster lung fibroblast cell line designated V79-S171-W1 was used in these experiments. The medium used was Basal Medium Eagle (BME) containing 13% heat-inactivated foetal calf serum and 1% penicillin and streptomycin from a stock solution $10^4 \mathrm{U\,ml^{-1}}$ and $10^4 \mu\mathrm{g\,ml^{-1}}$, respectively. During exponential growth, the cells had a population doubling time of $\sim$11 h. During the course of these experiments, V79 culture medium was changed to 1:1 Dulbecco's modified BME and F12 medium containing 5% foetal calf serum (DF5). Under these conditions the cells had the same doubling time as in BME but would grow at a much lower serum concentration. (The results of experiments done with DF5 medium are shown in Figures 6, 7 and 8). Cells were plated approximately 16 h before experimental procedures

© The Macmillan Press Ltd., 1983

were started in order to avoid changes in cellular sensitivity as a function of time after plating (Raaphorst *et al.*, 1979). At this time, cellular multiplicity ranged from 1.5–2.0 and multiplicity corrections were made according to standard procedures (Elkind and Whitmore, 1967).

The C3H mouse embryo cells developed by Reznikoff *et al.* (1973*a,b*) and designated 10T 1/2 clone 8 were obtained from the American Type Culture Collection. C3H-10T 1/2 cells were also obtained as a gift from Dr. J. Bedford. Cells were cultured at 37°C in BME containing 10% heat-inactivated foetal calf serum with no antibiotics. Under these conditions, the cell population doubling time during exponential growth phase was ~16–20 h. Cells were cultured up to passage 15 in Falcon T25 and T75 flasks; higher passage numbers were not used for experiments. Exponentially growing cells were then trypsinized, suspended as single cells in culture medium and replated in T25 flasks at the required cell numbers, 20–24 h before the experiments to obtain exponentially growing cells. At the beginning of the experiments, the cellular multiplicity ranged from 1.1–1.3 and multiplicity correction was made as noted above.

The morphologically transformed cell line was selected from a parent C3H-10T 1/2 cell population that had been exposed six weeks previously to $20\,\mu g\,ml^{-1}$ of 3-methylcholanthrene for 24 h. A cell colony with type-2 transformed morphology, as described by Reznikoff *et al.* (1973*a, b*) was removed from the monolayer using a sterile spatula. These cells were grown up to a monolayer and then reseeded into microtest dishes at a density of one cell per microwell. One single cell clone with the type-2 morphology was selected as the transformed cell line and labelled tr4. These cells, grown and propagated under conditions identical to those for the parent cell line, had a population doubling time of ~16–20 h. When these cells were grown to confluence and re-fed, they continued to grow into multi-layers and did not go into monolayer stationary phase as did the normal parent cells. Also, when the transformed cells were suspended in 1.5% methyl cellulose, growth and colony formation were observed. Thus, the cell line tr4 possessed properties typical of a transformed cell line as described by others (Reznikoff *et al.*, 1973; Borek, 1976; Sanford, 1975). However, the phenotypically transformed type II clones were shown to produce tumors 50–75% of the time when injected into mice (Reznikoff *et al.*, 1973,; Terzaghi and Little, 1976). The type II transformed cell line was not tested in animals for its tumorigenicity and consequently it can only be referred to as a morphological transformed cell line since all such cell lines will not produce tumours *in vivo*. The criteria of morphological transformation are routinely used in the literature since many studies have established the correlation between morphological transformation *in vitro* and tumorgenicity *in vivo* (Reznikoff *et al.*, 1973; Terzaghi and Little 1976).

Cells were cultured in Falcon T25 flasks and at the time of experimental treatment, flasks designated for heat exposure were sealed with wax. Heating was carried out in custom-designed water baths made from 0.5 inch Lucite. A Tempunit TU-14 thermoregulator (Techne, Princeton, NJ), was used for temperature regulation with additional stirring pumps. With this equipment, the temperature was maintained constant to ±0.02°C and was uniform throughout the bath to ±0.02°C. Temperature changes were made by transferring the flasks from one temperature-controlled bath to another. The half-time of temperature equilibration in a T25 flask containing 5 ml of medium was approximately 30 s.

Irradiation was carried out with a Siemens Stabilipan-2 X-ray machine operating at 250 kV and 15 mA with a 1-mm aluminium filter. The dose rate was $3.0\,Gy\,min^{-1}$ and the effective energy was 80 keV (HVL of 1.35 cm aluminium).

After 6–10 days for the V79 cells or 8–12 days for the C3H cells, the colonies within the flasks were rinsed, fixed with 95% ethanol, stained with methylene blue and counted. The survival data were corrected for the plating efficiency which ranged from 50–90% for the V79 cells and from 7–20% for the C3H-10T 1/2 cells.

For each experimental point, four replicate flasks were used and the standard error of the mean is indicated when greater than the size of the data point symbol. All experiments were repeated 2 or more times. The curves in the figures were fitted by eye. The thermal enhancement ratios (TER's) were calculated for the 10% survival level by taking the X-ray dose required to reduce survival to 10% and dividing by the X-ray dose required to reduce the survival of the cell population receiving hyperthermia to 10% of the survival level after heat treatment alone.

### Results

### *Thermal sensitivity, radiosensitization and recovery*

The thermal sensitivity of V79 and C3H-10T 1/2 cells is shown in Figure 1. The normal and transformed C3H cells were equally sensitive at 42.0°C and both these cell lines were more resistant than the V79 cells. At 42.0°C, the survival curves for the C3H and V79 cells were characterized by a thermotolerance plateau which commenced after about 4–6 h of heating. All the survival curves possessed a shoulder showing the capability for accumulation of sublethal damage. Other experiments (data not shown) show that at 43.0 and 45.0°C, the C3H-10T 1/2 cells were also more resistant than the V79 cells. The survival curve for V79 cells grown and heated at 42.0°C in DF5 medium (data not shown) was comparable to the curve for V79 treated in BME (Figure 1).

**Figure 1** Survival of V79 cells (●), C3H-10T 1/2 normal (■) and C3H-10T 1/2 cells transformed cells (□) after heating at 42.0°C.

**Figure 2** Survival of V79, normal C3H-10T 1/2 and transformed C3H-10T 1/2 cells for various sequences of heating and irradiation. The vertical bars represent the heating interval, the negative abscissa represents radiation before heating and the positive abscissa represents irradiation after heating. The time on the abscissa represents incubation time between treatments. Data points between the vertical bars are for simultaneous treatment and indicate the time of irradiation during heating. The diamonds represent data obtained from a different source of C3H-10T 1/2 cells (see **Materials and methods**). The survival levels for C3H-10T 1/2 normal cells after 1 or 1.5 h heating were 95% and 90%; for C3H-10T 1/2 transformed cells after 1.5 h heating was 80%; for V79 cells after 1.5 h or 3 h heating was 27 and 11%.

The responses of normal and transformed C3H-10T 1/2 cells and V79 cells to various sequences of hyperthermic and radiation exposure are shown in Figure 2. For V79 cells and normal and transformed 10T 1/2 cells, the greatest sensitivity occurred when the two treatments were administered simultaneously. The sequence dependence was greater for V79 cells than for 10T 1/2 cells. For both the treatment sequences of irradiation during or after heating, the transformed cell line was more sensitive to the combined treatments than the normal cell line. However, when cells were irradiated before heating, only a very small and insignificant difference was observed between the normal and transformed cell lines. Recovery was observed when 37°C incubation was carried out between the two treatments. When a shorter period of heat treatment (60 min at 42.0°C) and a smaller radiation dose (4.0 Gy) was used, the differences in sensitivity for irradiation before, during or after heating were not as large compared to the treatment of 90 min at 42.0°C and 6.0 Gy for the normal C3H-10T 1/2 cells. (The open and closed diamonds represent results obtained using the 10T 1/2 cells obtained from Dr. J. Bedford.) For all treatment sequences, even those separated by a 2 h incubation interval, a synergistic effect of heat and X-rays was observed for both the normal and the transformed cell lines.

*Thermotolerance and radiosensitization*

The thermal enhancement of radiosensitivity of both normal and transformed C3H-10T 1/2 cells was examined more extensively for heating at 42.0°C and the results are shown in (Figure 3). It should be noted that both the normal and transformed cells showed similar radio sensitivity (Figure 3) and heat sensitivity at 42.0°C (Figure 1). When hyperthermic treatment was completed 10 min before X-irradiation, the enhancement of radiosensitivity was greater in the transformed cell line compared to the normal cell line. The thermal enhancement ratios at 10% survival and the $D_0$s are given in Table I. For both the normal and transformed cell lines, thermal enhancement increased as the heating interval was prolonged. Even when thermotolerance developed after 6 h of heating, and little or no additional cell killing occurred at longer heating intervals, thermal radiosensitization was still increased for both the normal and the transformed cell lines.

Commencement of heating at 42.0°C, 10 min after irradiation or termination of heating at 42.0°C, 10 min before irradiation, resulted in enhanced radiosensitivity of V79 cells (Figures 4 and 5). Like the C3H cells, thermal radiosensitization increased in V79 cells as the heating time was increased to 7, 9 or 11 h even

**Figure 3** Survival after irradiation or heating at 42.0°C which was terminated 10 min before irradiation of C3H-10T 1/2 cells normal cells (closed symbols) or transformed cells (open symbols).

**Table I** Survival curve parameters for V79 cells heated and/or irradiated

| *Treatment** | $D_0(Gy)$ | *TER*† |
|---|---|---|
| X-rays | 1.61 | |
| 41.0°C (1.5 h) plus X-rays | 1.54 | 1.20 |
| 41.0°C (5 h) plus X-rays | 1.58 | 1.65 |
| 41.0°C (8 h) plus X-rays | 1.50 | 1.69 |
| 41.0°C (11 h) plus X-rays | 1.56 | 1.78 |
| X-rays plus 41.0°C (1.5 h) | 1.52 | 1.58 |
| X-rays plus 41.0°C (5 h) | 1.46 | 1.69 |
| X-rays plus 41.0°C (8 h) | 1.48 | 1.82 |
| X-rays plus 41.0°C (11 h) | 1.48 | 1.82 |
| X-rays | 1.43 | 1.0 |
| 42.0°C (1.5 h) plus X-rays‡ | 1.17 | 1.71 |
| 42.0°C (3.0 h) plus X-rays‡ | 1.17 | 2.32 |
| 42.0°C (5.0 h) plus X-rays‡ | 0.89 | 2.20 |
| 42.0°C (7.0 h) plus X-rays‡ | 0.83 | 2.36 |
| 42.0°C (9.0 h) plus X-rays‡ | 0.64 | 2.60 |
| 42.0°C (11.0 h) plus X-rays‡ | 0.52 | 2.82 |
| X-rays plus 42.0°C (1.5 h)‡ | 1.02 | 2.03 |
| X-rays plus 42.0°C (3.0 h)‡ | 0.91 | 2.76 |
| X-rays plus 42.0°C (5.0 h)‡ | 0.67 | 2.40 |
| X-rays plus 42.0°C (7.0 h)‡ | 0.62 | 2.50 |
| X-rays plus 42.0°C (9.0 h)‡ | 0.59 | 2.65 |
| X-rays plus 42.0°C (11.0 h)‡ | 0.52 | 2.82 |
| 45.0°C (20 min) plus X-rays | 0.52 | 4.31 |
| 45.0°C (10 min), 37°C (3 h), 45.0°C (10 min) plus X-rays | 0.91 | 2.76 |
| 45.0°C (10 min), 37°C (6 h), 45.0°C (10 min) plus X-rays | 0.95 | 2.30 |
| 45.0°C (10 min), 37°C (9 h), 45.0°C (10 min) plus X-rays | 1.30 | 2.09 |
| 45.0°C (10 min), 37°C (3 h), 45.0°C (25 min) plus X-rays | 0.54 | 5.75 |
| 45.0°C (10 min), 37°C (6 h), 45.0°C (25 min) plus X-rays | 0.84 | 3.29 |

*All heat and radiation exposures were separated by a 10 min incubation period at 37°C.

†Thermal enhancement ratio = dose to reduce survival to 10% for X-rays/X-ray dose to reduce survival to 10% of the survival after heating.

‡Results for cells that were grown and treated in BME while all other results are for cells grown and treated in DF 5.

though no additional killing was observed for heating done for intervals >5 h. The thermal enhancement ratios at the 10% survival level and the $D_0$s are given in Table I. At the shorter heating intervals, 1.5, 3.0, 5.0 or 7.0 hours, greater radiosensitization occurred when the cells were heated after irradiation, however, after 9.0 or 11.0 h of heating the difference in radiosensitivity was not very large for the two treatment protocols. Similar results were obtained for cells grown in DF5 medium and heated at 42.0°C (data not shown).

Further experiments were done to examine the effect of thermotolerance on thermal radiosensitization for V79 cells grown in DF5 medium. Figures 6 and 7 show the effect of heating at 41.0°C before or after irradiation on cellular radiosensitivity. Thermal radiosensitization is mainly characterized by decreases in the survival curve shoulders as heating at 41.0°C is prolonged either before or after irradiation. Only a small decrease is observed in the survival curve. $D_0$ (see Table I) which becomes constant for the longer heating intervals during which the cells attain a thermotolerant state. The thermal enhancement ratios calculated at the 10% survival level (Table I) show that little or no increase occurred in radiosensitization between 8 and 11 h of heating at 41.0°C during which time cells had become thermotolerant.

The radiosensitivity of cells developing thermotolerance after acute heating at 45.0°C is shown in Figure 8. The results in this Figure and the survival curve parameters shown in Table I indicate that radiosensitization by heating at 45.0°C is characterized by decreases in $D_0$. However thermal radiosensitization decreased when the heat treatment was divided into two equal fractions so that cells developed thermotolerance during incubation at 37°C between the heat treatments. Even when the second heat treatment was increased from 10–25 min cellular radiosensitivity was less for cells having received a 3 or 6 h incubation between the 10 and 25 min heat treatment compared to the cells having received a single heat treatment of 20 min.

The data presented in Tables I and II show the $D_0$s and the TERs at the 10% survival level of the survival curves shown in Figures 3–8. The $D_0$s for the survival curves for X-rays alone for the V79 and C3H-10T 1/2 cells were calculated for survival levels between 10% and 0.1% from additional data which are not shown in Figures 3 or 4. The $D_0$s for radiation survival data for V79 and C3H-10T 1/2 cells agree fairly well with some $D_0$s published in the literature (Raaphorst *et al.*, 1979; Terzaghi and Little, 1976; Raaphorst and Kruuv, 1977).

### Discussion

### *Thermal sensitivity, radiosensitization and recovery*

The heat sensitivity of V79 cells was greater than that of C3H-10T 1/2 cells as observed in Figure 1. The V79 cell line has a very short $G_1$ period compared to other cell lines (Raaphorst and Kruuv, 1977; Sinclair, 1972) and since $G_1$ cells are more heat resistant than S-phase cells (Westra and Dewey, 1971; Bhuyan *et al.*, 1977) the difference in age distribution of an asynchronous V79 or C3H-

**Figure 4** Effect of heating at 42.0°C on V79 cell radiosensitivity for heat treatments which terminated 10 min before irradiation, for the various heating intervals indicated on the figure.

10T 1/2 cell population may be involved in the observed differences in heat sensitivity.

The thermal radiosensitization was maximum for both V79 and C3H-10T 1/2 cells when simultaneously treated with heat and X-rays. This agrees with other studies using different cell lines (Sapareto *et al.*, 1978; Joshi *et al.*, 1978). In both the V79 and C3H-10T 1/2 cells, the recovery during an incubation interval at 37°C was larger when heat at 42.0°C followed X-rays, compared to X-rays following heat. This sequence dependent difference has also been observed for several other cell lines (Gerweck *et al.*, 1975; Joshi *et al.*, 1978). Larger heat treatments preceding irradiation resulted in reduced recovery during incubation at 37°C between heat and radiation treatments and the effect is potentiated when heating is done at acidic pH (Freeman *et al.*, 1981*a*, *b*). These data imply that heat treatments which result in a large amount of heat damage, inhibit or damage the repair mechanisms and consequently prevent recovery at 37°C of the heat induced damage which can interact with subsequent radiation induced damage. The results in Figures 4 and 5 also support this conclusion in that thermal sensitization was greater for heating after irradiation for heating times of $<7$ h while at longer heating times thermal radiosensitization was about the same for heating before or after irradiation.

The sequence-dependent difference in recovery was much larger in V79 cells compared to C3H-10T 1/2 cells. The rapid and large recovery during incubation, when heat followed radiation compared to the relatively small and slow recovery when

**Figure 5** Effect of heating at 42.0°C on V79 cell radiosensitivity for heating commencing 10 min after irradiation, for the various heating intervals as indicated on the figure.

**Figure 7** Effect of heating at 41.0°C on radiosensitivity of V79 cells cultured in DF5 medium. Heat treatments were started 10 min after irradiation.

**Figure 6** Effect of heating at 41.0°C on radiosensitivity of V79 cells cultured in DF5 medium. Heat treatments were terminated 10 min before irradiation.

**Table II** Survival curve parameters for C3H-10T 1/2 cells heated and/or irradiated

| *Treatment** | *Cell line* | $D_0(Gy)$ | *TER†* |
|---|---|---|---|
| X-rays | C3H-10T 1/2 N‡ | 1.86 | 1.0 |
| 42.0°C (2.5 h) plus X-rays | C3H-10T 1/2 N | 1.19 | 1.80 |
| 42.0°C (6.0 h) plus X-rays | C3H-10T 1/2 N | 1.04 | 1.89 |
| 42.0°C (11.0 h) plus X-rays | C3H-10T 1/2 N | 0.58 | 2.24 |
| X-rays | C3H-10T 1/2 T§ | 1.86 | 1.0 |
| 42.0°C (2.5 h) plus X-rays | C3H-10T 1/2 T | 0.93 | 2.05 |
| 42.0°C (6.0 h) plus X-rays | C3H-10T 1/2 T | 0.89 | 2.33 |
| 42.0°C (11.0 h) plus X-rays | C3H-10T 1/2 T | 0.45 | 2.69 |

*All heat and radiation exposures were separated by a 10 min incubation period at 37°C.

†Thermal enhancement ratio = dose to reduce survival to 10% for X-rays/X-ray dose to reduce survival to 10% of the survival after heating.

‡C3H-10T 1/2 normal cell line.

§C3H-10T 1/2 MCA transformed cell line.

**Figure 8** Effect of heating at 45.0°C on radiosensitivity of V79 cells grown in DF5 medium. Heat exposures were given as single or split treatments separated by incubation at 37°C and irradiation was done 10 min after the last heat treatment.

radiation followed heat, implies that the two types of lesions are different. It is important to note that in V79 cells, heat treatment immediately before irradiation resulted in higher survival than heat immediately after irradiation while in the C3H-10T 1/2 cells the opposite was true. Such cell-line dependence on treatment sequences has also been observed by others (Li and Kal, 1977). These cell-type-dependent differences of heat and radiosensitivity and the dependence on treatment sequence may have important clinical implications for therapy.

Even though the heat and radiation sensitivity of the normal and transformed C3H-10T 1/2 cells were approximately the same, sensitivity to the combined treatments was different. The transformed cells were more sensitive to heat plus radiation than the normal cells for several heat-treatment intervals at 42.0°C. This difference was observed for the simultaneous treatment or heat treatment before irradiation while for heat treatment after irradiation very little difference was observed. These data imply that the fixation of radiation-induced lesions by heating after irradiation was the same in both cell lines while the effect of heating before irradiation may have a differential effect on the repair capacity of the normal and transformed cell lines. Thus, even though the heat and X-ray sensitivities of a normal and transformed cell line may be the same, their response to the combined treatment can be different and, consequently, result in therapeutic gain, if this phenomenon occurs *in vivo* as well as *in vitro*.

*Thermal tolerance and radiosensitization*

Both the V79 and C3H cell lines developed thermotolerance after continuous heating at 42.0°C. Even though the V79 cells were more heat sensitive than the C3H-10T 1/2 cells, thermotolerance set in at about the same time (4–6 h) which is consistent with the data in the literature (Dewey *et al*., 1979; Raaphorst *et al*., 1979).

The thermal radiosensitization increased as a function of heating time at 42.0°C for V79 cells and normal or transformed C3H-10T 1/2 cells. Even when the cells attained thermotolerance after a 4–6 h heating interval and no further cell killing occurred, additional radiosensitization resulted from the longer heating intervals. Thermal radiosensitization was greater in V79 cells heated for 11 h before irradiation compared to C3H-10T 1/2 cells. This agrees with observations in an earlier study (Raaphorst *et al*., 1979) that cells with a greater thermal sensitivity are more radiosensitized by thermal treatment.

In a study by Freeman *et al.* (1979) it was found that in CHO cells thermal radiosensitization did not increase with increased heating time at 42.0°C after they have attained a thermotolerant state while the results of Holahan *et al.* (1982) show that there was an actual decrease in thermal radiosensitization as cells attained thermal tolerance. Since these results differed from the results with V79 cells and C3H-10T 1/2 cells we further investigated this phenomenon at a lower temperature such as 41.0°C. The data show that as V79 cells reach a thermotolerant state during 41°C heating, radiosensitivity is not further increased by additional heating. This result occurs for both heating before or after irradiation and demonstrates that V79 cells display similar characteristics with respect to radiosensitivity and thermotolerance as CHO cells except the effect occurs at a lower temperature. Such a temperature threshold may be cell line dependent.

Cells also develop thermotolerance when the heating interval is split into 2 fractions and incubation at 37°C is carried out between the two heat treatments. The results presented in Figure 8 show that thermal radiosensitization decreases as cells become thermotolerant. The data of Henle *et al.* (1979) show no change in radiosensitivity for heating 24 h before irradiation but show a decrease in thermal radiosensitization for heating 24 h before a subsequent heat and X-ray treatment. The results of Nielson and Overgaard (1979) do not agree with the above in that they show no change in thermal enhancement ratio (TER) in the presence or absence of a conditioning heat treatment 10 h before heating and irradiation. However, their data were complicated by the fact that the conditioning heat treatment 10 h before irradiation caused the survival curve $D_0$ to increase from 1.17 to 1.42 Gy which would result in a larger calculated TER. Further results of Miyakoshi *et al.* (1979) showed that radiosensitization was diminished if low temperature hyperthermia (42.0°C) preceded high temperature hyperthermia at 45.0°C causing thermotolerance. Results from normal tissue studies *in vivo* also show that a conditioning heat treatment given at various times before subsequent heating and irradiation could result in thermal tolerance and reduced TERs (Henle 1982, Law *et al.*, 1979*a,b,* Dethlefsen and Dewey 1982). In these studies it was shown that the reduction in TERs was not temporally related to the onset of thermal tolerance (Marigold and Hume, 1982; Law *et al.*, 1979*a,b*). Our data showed a temporal correlation between the onset of thermal tolerance and the decrease in thermal enhancement of radiosensitivity. Likewise, thermotolerance and decreased thermal radiosensitization are also correlated in other studies of (Holahan *et al.*, 1982; Miyakoshi *et al.*, 1979 and Henle *et al.*, 1979). All these results indicate that thermal tolerance in radiosensitization can also occur and these results correlate with the onset of thermotolerance *in vitro*.

Since differences in the *in vivo* and the *in vitro* results exist it is necessary to examine the induction of thermotolerance and reduced thermal radiosensitization in more detail in more mammalian cell and tissue systems to determine whether these two processes occur by similar or different mechanisms. It may be that *in vivo*, physiological parameters could be altered by the conditioning heat treatment to cause a subsequent change in radiosensitivity which is not temporally correlated with the onset of thermal tolerance.

### References

BOREK, C. (1976). *In vitro* transformation by low doses of X-irradiation and neutrons. In *Biology of Radiation Carcinogenesis* (Eds. J.M. Juhas, R.W. Tennant & J.D. Regan). Raven Press, New York.

BHUYAN, B.K., DAY, K.J., EDGERTON, C.E. & OGUNBASE, O. (1977). Sensitivity of different cell lines and different phases in the cell cycle to hyperthermia. *Cancer Res.*, **37**, 3780.

CONNOR, W.G., GERNER, E.W., MILLER, R.C. & BOONE, M.L.M. (1977). Prospects for hyperthermia in human cancer therapy II, Implications of biological and physical data for applications of hyperthermia to man. *Radiol.*, **123**, 497.

DEWEY, W.C., FREEMAN, M.L., RAAPHORST, G.P. & 7 others. (1978). Cell biology of hyperthermia and radiation. In *Radiation Biology in Cancer Research.* (Eds. R.E. Meyn & H.R. Withers) p. 589. Raven Press: New York.

DETHLEFSEN, L.A. & DEWEY, W.C. (Eds) (1982). Proc. Third Int. Symp Cancer Therapy by Hyperthermia Drugs and Radiation. *NCI Monograph,* **61,** p. 279, 283, 291.

ELKIND, M.M. & WHITMORE, G.F. (1967). *The Radiobiology of Cultured Mammalian Cells.* p. 1 Gordon and Breach, New York.

FREEMAN, M.L., HOLAHAN, E.V., HIGHFIELD, D.P., RAAPHORST, G.P., SPIRO, I.J. & DEWEY, W.C. (1981*a*). The effect of pH on hyperthermic and X-ray induced cell killing. *Int. J. Radiat. Oncol. Biol. Phys.*, **1**, 211.

FREEMAN, M.L., BOONE, M.L., ENSLEY, B.A. & GILLETTE, E.L. (1982*b*). The influence of environmental pH on the interactions and repair of heat and radiation damage. *Int. J. Radiat. Oncol.* (In press).

FREEMAN, M.L., RAAPHORST, G.P. & DEWEY, W.C. (1979). The relationship of heat killing and thermal radiosensitization to the duration of heating at 42.0°C. *Radiat. Res.*, **78**, 172.

GERWECK, L.E., GILLETTE, E.L. & DEWEY, W.C. (1975). Effect of heat and radiation on synchronous Chinese hamster cells: Killing and repair. *Radiat. Res.*, **64**, 611.

GERWECK, L.E. & BURLETT, P. (1978). The lack of correlation between heat and radiation sensitivity in mammalian cells. *Int. J. Radiat. Oncol.*, **4**, 283.

GIOVANELLA, B.C., STEHLIN, J.S. & MORGAN, A.C. (1976). Selective lethal effect of supranormal temperatures on human neoplastic cells. *Cancer Res.*, **36**, 3944.

HARISIADIS, L., HALL, E.J., KARLJEVIC, U. & BOREK, C. (1975). Hyperthermia: Biological studies at the cellular level. *Radiology*, **117**, 447.

HENLE, K.J. (1982). Thermotolerance in the murine jejunum, *J. Natl Cancer Inst.*, **68**, 1033.

HENLE, K.J., TOMASOVIC, S.P. & DETHLEFSEN, L.A. (1979). Fractionation of combined heat and radiation in asynchronous CHO cells, 1. Effects on radiation sensitivity. *Radiat. Res.*, **80**, 369.

HOLAHAN, E.V., HIGHFIELD, D.P., STUART, P.K. & DEWEY, W.C. (1982). Hyperthermic killing and hyperthermic radiosensitization in CHO cells. *Radiat. Res.*, **91**, 313.

JOSHI, D.S., BARENDSEN, G.W. & VANDER SCHUEREN, E. (1978). Thermal enhancement of the effectiveness of gamma radiation for induction of reproductive death in cultured mammalian cells. *Int. J. Radiat. Biol.*, **34**, 233.

KASE, K. & HAHN, G.M. (1975). Differential heat response of normal and transformed human cells in tissue culture. *Nature*, **255**, 228.

LI, G.C. & KAL, H.B. (1977). Effect of hyperthermia on the radiation response of two mammalian cell lines. *Europ. J. Cancer*, **13**, 65.

LAW, M.P., COULTAS, P.G. & FIELD, S.B. (1979*a*). Induced thermal resistance in the mouse ear. *Br. J. Radiol.*, **52**, 308.

LAW, M.P., AHIER, R.G. & FIELD, S.B. (1979*b*). The effect of prior heat treatment on thermal enhancement of radiation damage in the mouse ear. *Br. J. Radiol.*, **52**, 315.

MARIGOLD, J.C.L. & HUME, S.P. (1982). Effect of prior hyperthermia on subsequent thermal enhancement of radiation damage in mouse intestine. *Int. J. Radiat. Biol.*, **42**, 509.

MIYAKOSHI, J., IKEBUSHI, M., FURUKAWA, M., YAMAGATA, K., SUGAHARA, T. & KANO, E. (1979). Combined effects of X-irradiation and hyperthermia (42 and 44°C) on Chinese hamster V-79 cells *in vitro*. *Radiat. Res.*, **79**, 77.

MUCKLE, D.S. & DICKSON, J.A. (1971). The selective inhibitory effect of hyperthermia on the metabolism and growth of malignant cells. *Br. J. Cancer*, **25**, 771.

NIELSON, O.S. & OVERGAARD, J. (1979). Hyperthermic radiosensitization of thermotolerant tumor cells *in vitro*. *Int. J. Radiat. Biol.*, **35**, 171.

OVERGAARD, J. (1977). Effect of hyperthermia on malignant cells *in vivo*. *Cancer*, **39**, 2637.

RAAPHORST, G.P. & AZZAM, E.I. (1980). Dependence of heat and X-ray sensitivity of V79 cells on growth media and various serum combination. *Int. J. Radiat. Biol.*, **38**, 667.

RAAPHORST, G.P., FREEMAN, M.L. & DEWEY, W.C. (1979). Radiosensitivity and recovery from radiation damage in cultured CH0 cells exposed to hyperthermia at 42.5 or 45.5°C. *Radiat. Res.*, **79**, 390.

RAAPHORST, G.P., ROMANO, S.L., MITCHELL, J.B., BEDFORD, J.S. & DEWEY, W.C. (1979). Intrinsic differences in heat and/or X-ray sensitivity of seven mammalian cell lines cultured and treated under identical conditions. *Cancer Res.*, **39**, 396.

RAAPHORST, G.P., SAPARETO, S.A., FREEMAN, M.L. & DEWEY, W.C. (1979). Changes in cellular heat and/or radiation sensitivity observed at various times after trypsinization and plating. *Int. J. Radiat. Biol.*, **35**, 193.

RAAPHORST, G.P. & KRUUV, J. (1977). Effect of salt solutions in radiosensitivity of mammalian cells. II Treatment with hypotonic solutions. *Int. J. Radiat. Biol.*, **32**, 89.

REZNIKOFF, C.A., BRANKOW, D.W. & HEIDELBERGER, C. (1973*a*). Establishment and characterization of a cloned line of C3H mouse embryo cells sensitive to postconfluence inhibition of division. *Cancer Res.*, **33**, 3231.

REZNIKOFF, C.A., BERTRAM, J.S., BRANKOW, D.W. & HEIDELBERGER, C. (1973*b*). Quantitative and qualitative studies of chemical transformation of cloned C3H mouse embryo cells sensitive to postconfluence inhibition of cell division. *Cancer Res.*, **33**, 3239.

SANFORD, K.K. (1975). Biological manifestations of oncogenesis *in vitro*: A Critique. *J. Natl Cancer Inst.*, **55**, 1481.

SAPARETO, S.A., HOPWOOD, L.E. & DEWEY, W.C. (1978). Combined effects of X-irradiation and hyperthermia on CHO cells for various temperatures and orders of application. *Radiat. Res.*, **73**, 221.

SINCLAIR, W.K. (1972). Cell cycle dependence of the lethal radiation response in mammalian cells. *Current Topics in Radiat. Res. Quart.*, **7**, 264.

STREFFER, C., VAN BEUNINGEN, D., DIETZEL, F. & 5 others. (1978). Proceedings of the 2nd International Symposium and Cancer Therapy by Hyperthermia and Radiation. Urban and Schwarzenberg, Baltimore-Munich.

TERZAGHI, M. & LITTLE, J.B. (1976). X-radiation-induced transformation in a C3H mouse embryo derived cell line. *Cancer Res.*, **36**, 1367.

WESTRA, A. & DEWEY, W.C. (1971). Variation in sensitivity to heat shock during the cell cycle of Chinese hamster cells *in vitro*. *Int. J. Radiat. Biol.*, **19**, 467.